# Assessment of Vegetation Habitat Types and Characteristics of Potential Nesting Trees for White‐Backed Vultures ( *Gyps africanus* ) in Kempiana and Manyeleti Nature Reserves, Mpumalanga Province, South Africa

**DOI:** 10.1002/ece3.71147

**Published:** 2025-03-20

**Authors:** Fidèle Ezéchiel K. Hounnouvi, Stanislas Mahussi Gandaho, Fern Bain, Peter Hamming, Lindy J. Thompson

**Affiliations:** ^1^ University of Kinshasa, ERAIFT Kinshasa Democratic Republic of the Congo; ^2^ University of Abomey‐Calavi Laboratory of Applied Ecology Cotonou Benin; ^3^ Southern African Wildlife College Springvalley Farm 200KU Kempiana Nature Reserve Hoedspruit South Africa; ^4^ Centre for Functional Biodiversity, School of Life Science University of KwaZulu‐Natal Pietermaritzburg South Africa

**Keywords:** GIS, modeling, nesting sites, raptors, vulture, White‐backed Vulture, modélisation, rapaces, SIG, sites de nidification, vautour, vautour à dos blanc

## Abstract

The White‐backed Vulture ( *Gyps africanus* ) is widespread in sub‐Saharan Africa but faces population decline due to habitat degradation, poisoning, food loss, and energy infrastructure. This study examines how tree species, height, trunk circumference, and vegetation type influence nest placement in the Kempiana and Manyeleti Nature Reserves, South Africa. Using high‐resolution aerial imagery, we identified habitat types and surveyed 106 trees, including 31 nesting trees. Data collection spanned 3 weeks (12.02.2024–01.03.2024) during the non‐breeding season, with fieldwork conducted 5 days per week (6 h/day). A four‐ six‐member team recorded geomorphological variables via GIS and tree characteristics. The thinning vegetation in autumn enhanced nest visibility. Nesting trees were found across four vegetation types: 67% in mixed woodland with grass, 20% in wooded savanna, 10% in savanna grassland, and 3% in bare soil/built areas. Fischer's exact test and Dunn's test indicated that nesting trees were significantly selected based on height, trunk circumference, and proximity to rivers and roads. Nesting trees had a median height of 14.5 m, a CBH of 2.12 m, and were located a median distance of 2.23 km from rivers and 5.65 km from roads. Our findings confirm that White‐backed Vultures prefer large trees near rivers but away from roads for nesting. We recommend future research on habitat degradation, particularly the loss of large riparian trees, and its impact on nesting site availability. Conservation efforts should focus on preserving large trees and reducing anthropogenic disturbances in critical nesting areas.

## Graphical abstract

The study revealed that Scavenger Vultures ( *Gyps africanus* ) prefer to nest in mixed forests with grass rather than in grasslands, bare ground, or built‐up environments. They also choose tall, wide trees. These results confirm that these vultures do not choose their nesting trees at random, but according to specific criteria. Further research is needed to assess the areas suitable for nesting by these vultures.

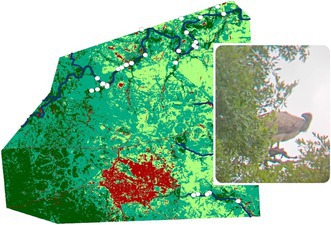

## RESUME

Le vautour à dos blanc ( *Gyps africanus* ) est largement répandu en Afrique subsaharienne, mais sa population est confrontée à un déclin dû à la dégradation de l'habitat, à l'empoisonnement, à la perte de nourriture et à l'infrastructure énergétique. Cette étude examine comment les espèces d'arbres, la hauteur, la circonférence du tronc et le type de végétation influencent le placement des nids dans les réserves naturelles de Kempiana et de Manyeleti, en Afrique du Sud. À l'aide d'images aériennes à haute résolution, nous avons identifié les types d'habitats et recensé 106 arbres, dont 31 arbres de nidification. La collecte des données s'est étalée sur trois semaines (12.02.2024–01.03.2024) pendant la saison de non‐reproduction, avec un travail de terrain effectué cinq jours par semaine (6 heures/jour). Une équipe de quatre à six membres a enregistré les variables géomorphologiques via le SIG et les caractéristiques des arbres. L'éclaircissement de la végétation en automne a amélioré la visibilité des nids. Les arbres nicheurs ont été trouvés dans quatre types de végétation: 67% dans des forêts mixtes avec de l'herbe, 20% dans des savanes boisées, 10% dans des prairies de savane, et 3% dans des sols nus/zones construites. Le test exact de Fischer et le test de Dunn ont indiqué que les arbres de nidification étaient sélectionnés de manière significative en fonction de la hauteur, de la circonférence du tronc et de la proximité des rivières et des routes. Les arbres de nidification avaient une hauteur médiane de 14,5 m, une circonférence du tronc de 2,12 m, et étaient situés à une distance médiane de 2,23 km des rivières et de 5,65 km des routes. Nos résultats confirment que les vautours à dos blanc préfèrent les grands arbres près des rivières mais loin des routes pour nicher. Nous recommandons des recherches futures sur la dégradation de l'habitat, en particulier la perte de grands arbres riverains, et son impact sur la disponibilité des sites de nidification. Les efforts de conservation devraient se concentrer sur la préservation des grands arbres et la réduction des perturbations anthropogéniques dans les zones de nidification critiques.

## 1 Introduction

Vultures are an important part of the ecosystems they inhabit, playing a vital role in cleaning up the environment (Ogada et al. 2012) and likely reducing the spread of disease (BirdLife International 2020; IUCN Vulture Specialist Group 2020). These ecosystem services have an impact on the quality of the environment, health, and socio‐economic conditions for humans (Markandya et al. 2008; Frank and Sudarshan 2024). Hence, the population declines of vultures have severe ecological and socio‐economic implications (Pain et al. 2003; Markandya et al. 2008), and yet, of the 23 species of vultures worldwide, 16 are listed as threatened (IUCN 2021; Buechley and Şekercioğlu 2016).

Indeed, *two primary drivers of these declines are the deliberate and accidental poisoning of vultures, along with the unsustainable harvesting of these birds driven by cultural practices and the fetish trade* (Ogada et al. 2016). The habitat loss and degradation that follows closely result mainly from the conversion of grazing land to crops (Virani et al. 2011), encroachment of scrubland (Schultz 2007) and destruction of large trees by elephants.

The consequences of these declines for ecosystems are potentially significant because vultures are the only known obligate scavengers among the terrestrial vertebrates (DeVault et al. 2016). The White‐backed Vulture ( *Gyps africanus* ) is critically endangered, with a high risk of extinction (BirdLife International 2021).

Vultures can move large distances each day in response to the high degree of spatial and temporal variation in their food resources (Murn et al. 2013). During the breeding season, however, breeding adults need to concentrate their activities around their nesting sites (Bamford et al. 2007). While this may simplify their protection locally during the breeding period, their extensive movements outside of the breeding season and the potential variability in habitat requirements across different regions introduce additional challenges for their conservation. White‐backed Vultures have a long breeding cycle (6–9 months) and spend much of each year near their nesting sites. Understanding these dynamics is crucial for effective conservation planning. White‐backed Vultures have a long breeding cycle (6–9 months) and thus spend much of each year near their nest sites.

Identification of successful' nesting sites is often critical for the conservation of raptor species (Donazar et al. 2002), but such assessments are not feasible without a detailed understanding of the species' nesting requirements (Sara and Di Vittorio 2003). Successful habitat needs to be identified and then receive adequate protection by the development of effective in situ conservation strategies (Anoop et al. 2020). More detailed knowledge about successful nesting sites can help to determine potential habitats for protection. Investigating the relationship between the distribution of vulture nests and vegetation habitat type is therefore vitally important (Monadjem and Garcelon 2004).

The findings of this study could serve as a reference for protected area managers and conservation practitioners in the region, helping them recognize key nesting habitats and implement appropriate protective measures. Rather than directly advocating for the establishment of new protected areas, this research seeks to inform targeted conservation actions such as seasonal protection of nesting sites and safeguarding of key nesting trees in nest monitoring programs. In this context, our study aims to understand and identify White‐backed Vulture habitat preferences and nesting requirements to facilitate the conservation of the species in the Kempiana and Manyeleti Nature Reserves in north‐eastern South Africa.

This study has two specific objectives: (i) To identify different vegetation habitat types (e.g., savanna, forest, grassland) in the reserves to assess whether the distribution of nesting trees is associated with vegetation type, and (ii) to characterize nesting and non‐nesting trees to determine whether nest placement is associated with specific tree species/characteristics.

## 2 Methodology

### 2.1 Study Area

Our research took place in both Kempiana Nature Reserve and Manyeleti Nature Reserve, in South Africa's Mpumalanga province, over an area of 223 km^2^ (22,300 ha). Since 1990, the World‐Wide Fund for Nature (WWF‐SA) has owned Kempiana, and it is overseen by the Kempiana Management Committee. It is situated directly to the west of the Orpen Gate of the Kruger National Park (KNP) and to the south of Timbavati Private Nature Reserve.

**FIGURE 1:**
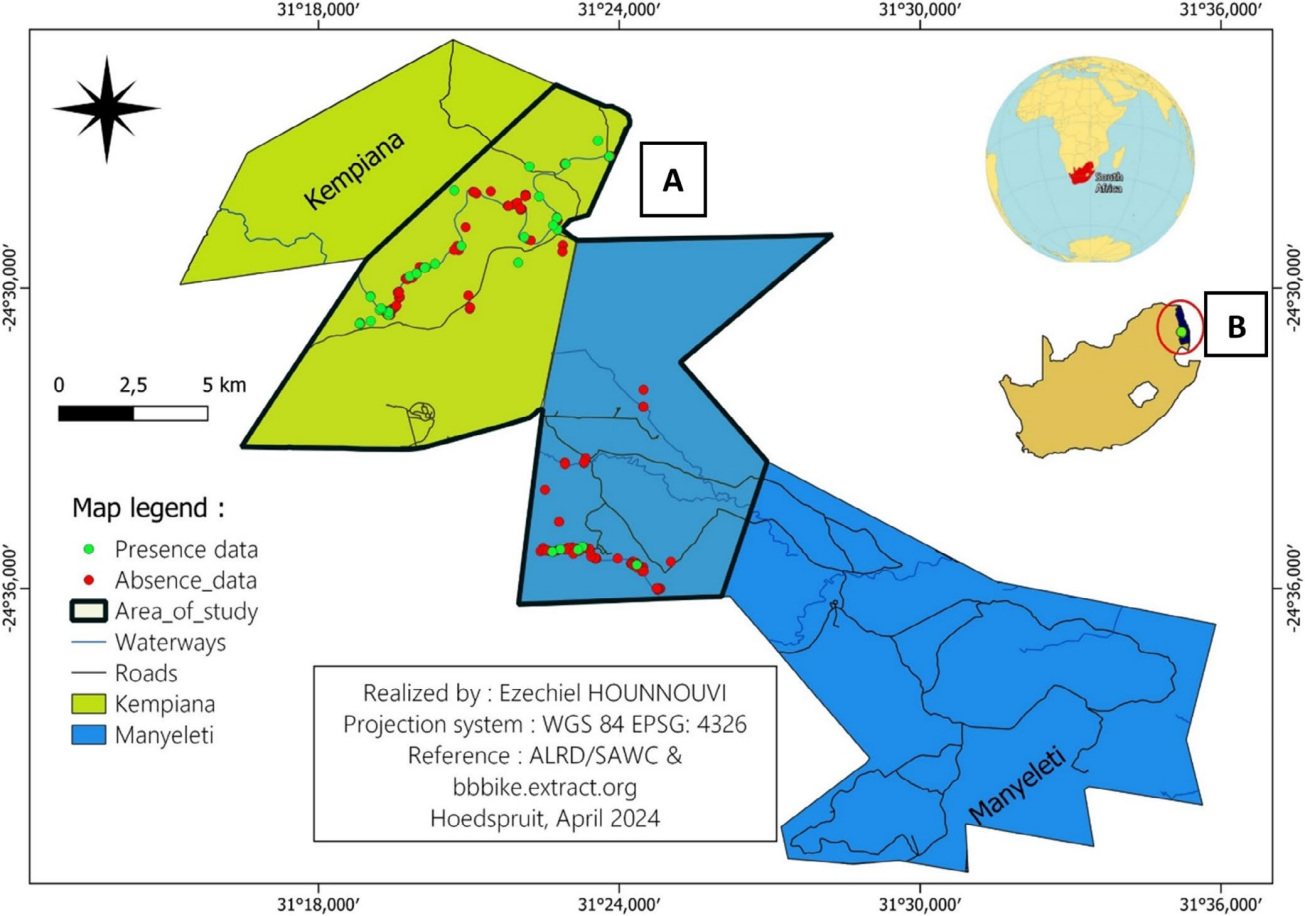
(A) The study area, comprising parts of Kempiana Nature Reserve (in green) and Manyeleti Nature Reserve (in blue), in Mpumalanga Province, South Africa. The area of interest is bordered by a black line. (B) The study area (green point) is shown in relation to Kruger National Park (black).

The Manyeleti Nature Reserve was established in 1967 in a region that was primarily utilized for raising cattle (Balyamujura and Van Schalkwyk 1999). Manyeleti Nature Reserve is southwest of KNP's Orpen Gate, in the lowveld savanna of South Africa. The reserve was originally comprised of several private reserves, separated by fences, until the land came under the management of the Mpumalanga Tourism and Parks Agency (MTPA) after 1994 (Marshal et al. 2012) (Figure 1).

### 2.2 Selection of Vulture Nesting Trees

White‐backed Vulture nesting trees were identified through intensive field surveys along rivers and their tributaries, using historical GPS data provided by and Beyond Ngala's ecological monitoring team. This team had previously recorded the locations of 16 nesting trees. During our fieldwork, we used these coordinates to relocate and record these trees if nests are still present (active or not) and to check if there were fresh molted feathers under the trees. Additionally, we identified and recorded 15 other nesting trees encountered during our surveys. Since our study took place outside the breeding season, we could not determine nest activity at the time of observation. All 31 nesting trees with nests, occupied or unoccupied but with evidence of occupation in recent years, were studied (Gavashelishvili et al. 2006). However, our focus was on identifying trees that had been selected by vultures for nesting, regardless of whether the nests were currently active, abandoned, or successful. We walked close to the rivers as well as far from them, following the lines of the rivers. The river lines served as a sort of compass because this vulture species is known to nest along rivers (Mundy et al. 1992).

### 2.3 Selection of Non‐Nesting Trees

We chose to use the minimum White‐backed Vulture nest tree measurements mentioned in the scientific literature as our minimum criteria for selecting on‐nesting trees. These included Circumference at Breast Height (CBH) and tree height.

These trees are selected randomly across the entire study area, regardless of nest presence. They are chosen based on the nesting trees minimum size criteria, which make them potential nesting tree and their location is independent of nest sites. We located a selection of tall trees using aerial imagery from the South African Environmental Observation Network (SAEON) using the random coordinate generator Inside Polygon tool in QGIS software (Johnson and Murn 2023). We subtracted the Digital Terrain Model (DTM) from the Digital Surface Model (DSM) using the Raster calculator tool in QGIS to obtain the Normalized Digital Surface Model (height) of the trees in our study area. This enabled us to randomly select and extract the GPS coordinates of 75 trees with a minimum height of 8 m. Then, in the field, we used a GPS to find them, checked that they did not contain nests and respect the minimum criteria (height ≥ 8 m and CBH ≥ 140 cm), then we took measurements. This procedure has been applied in other raptor habitat studies (Suárez et al. 2000).

### 2.4 Field Data Collection

Fieldwork was conducted from 12/02/2024 to 01/03/2024, outside of the breeding season of White‐backed Vultures and other resident vulture species in our study area, to avoid any disturbance to breeding birds (Van Stuyvenberg et al. 2014). Fieldwork was conducted by teams of four to six people, usually starting at around 08:30 and ending at around 14:30. Since we were operating in big‐five nature reserves, we were accompanied by an armed guard at all times.

During surveys, data were collected on the characteristics of each tree (Table 1). We confirmed the tree species names using a Field Guide to trees of Southern Africa book (Van Wyk 2013). We measured the circumference of each tree's trunk at two different heights (at 1.3 m to conform with international standards and at 0.3 m above the ground to conform with South African methods). We used a measuring tape for CBH measurements, and we estimated tree height against a 10 m pole held vertically close to the trunk of the tree. We also measured canopy width at the widest point of the canopy using a tape measure.

**TABLE 1:** Variables collected at vulture nest trees, control trees, and random trees, in Kempiana and Manyeleti Nature Reserves, in February 2024.

Variable	Definition (units)
**At the tree level**	
Tree species	The tree species name, confirmed using a fieldguide (Van Wyk 2013).
Tree height	Tree height (m). Measured using graduated poles held vertically against the tree trunk.
Tree circumference (CBH 1 and CBH 2)	Two circumferences were measured on the tree trunk: (i) CBH1 at breast height (1.3 m above the ground), and (ii) CBH2 at 0.3 m above ground level.
Canopy width	Canopy width at its widest point (m) using a measuring tape held taut by two researchers.
Number of nests	Number of White‐backed Vulture nests in the tree (N).
**At the habitat level**	
Digital Terrain Model (DTM)	Meters above sea level using DTM from SAEON aerial imagery (m). *Source:* SAEON (https://doi.org/10.15493/SAEON.NDLOVU.220424)
Normalized Digital Surface Model (NDSM)	Normalized Digital Surface Model (m). Result obtained by extracting the DTM from the DSM. *Source:* SAEON (https://doi.org/10.15493/SAEON.NDLOVU.220424). This method provides the height of trees with an accuracy of 92.9% compared to terrestrial measurements (Zimmermann and Hoffmann 2017).
Slope	The degree of incline of a surface (%). The slope was downloaded from opentopography.org platform (https://doi.org/10.5069/G9028PQ) and then cropped to our study area. Slope Values were extracted using the ‘Extractor’ plugin in QGIS.
VegType	Type of vegetation, obtained after a supervised classification of the aerial image.
Distance to river	Distance from tree to nearest river (km). *Source:* https://download.bbbike.org/osm/extract/planet_30.89,‐24.886_31.746,‐24.287.osm.shp.fr.zip
Distance to road	Distance from tree to nearest tar road, dirt road or railway line (km).
	Source: https://download.bbbike.org/osm/extract/planet_30.89,‐24.886_31.746,‐24.287.osm.shp.fr.zip
Distance to building	Distance from tree to nearest area of human activity (e.g., homes, offices, factories etc.) in km.
	Source: https://download.bbbike.org/osm/extract/planet_30.89,‐24.886_31.746,‐24.287.osm.shp.fr.zip

### 2.5 GIS Data Collection

At the habitat level, we collected variables related to geomorphology; Normalized Digital Surface Model (NDSM), Digital Terrain Model (DTM), ‘Slope’, and ‘Distance to River’. We also recorded ‘vegetation type’, along with ‘Distance to Roads’ and ‘Distance to Buildings’ as proxies for disturbance. These variables were obtained from the Geographical Information System (GIS). To get measures for the variables (Distance to Rivers, Distance to Roads, and Distance to Buildings), shapefiles of rivers, roads and buildings were downloaded from https://extract.bbbike.org/ which we clipped to our study area. These data were converted to raster format using QGIS software, and we used the “proximity distance raster” function in combination with the QGIS “Extractor” plugin to extract distance measurements from each sampled tree to the nearest rivers, roads, and buildings.

Aerial images acquired by SAEON were used to classify the vegetation of Kempiana and Manyeleti Nature Reserves. The images contained some metadata, including DTM, DSM, and slope. These metadata allowed us to obtain the NDSM (which meant that height was obtained by subtracting the DTM from the DSM), the DTM (altitude) and the slope. The aerial image from SAEON was acquired by flying at an altitude of 2000 m and shooting with an ILCE‐7RM4, FE 55 mm F1.8 at a 14 cm resolution. A set of GPS points representative of the entire reserve was collected in all vegetation types (Dossa et al. 2021) for validation.

After data collection, we processed the data in R (Team 2000). We imported data from multiple Excel sheets with the *readxl* R package (Wickham et al. 2019), standardized column names, and combined the datasets.

### 2.6 Data Analyses

#### 2.6.1 Identification of Vegetation Types in Kempiana and Manyeleti Nature Reserves

**FIGURE 2:**
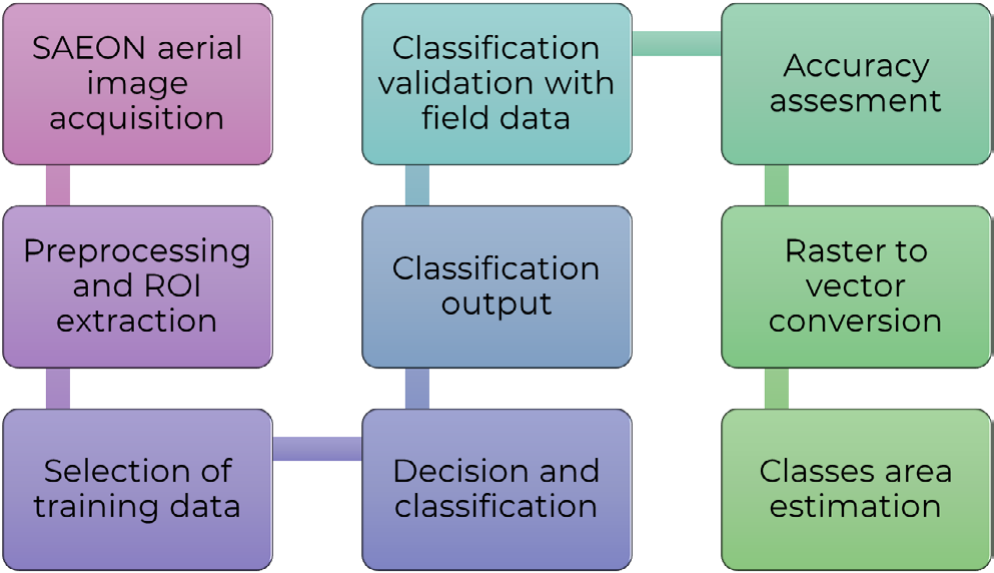
Steps in the classification of aerial imagery.

To achieve our objective of identifying vegetation types in our study area, we applied supervised classification (Figure 2). This choice was motivated by the knowledge of the region of interest, the accuracy of SAEON's aerial images, and the reliability of the method (Salata 2021).

To determine vegetation types by using image classification, we first extracted the study area from the aerial image. We performed a supervised classification (Figure 2) using the Maximum Likelihood algorithm based on training points collected in the field (Brun et al. 2018). We used the SCP plugin in QGIS, and the confusion matrix and the Kappa coefficient were determined to ensure accurate classification. Savanna vegetation and Mixed woodlands with grass vegetation habitats were a little similar, but we separated them based on the tree canopy cover. Savanna vegetation comprises a continuum of vegetation types with varying degrees of canopy cover. Mixed woodland with grass comprises more trees than wooded savanna. The key difference lies in tree and grass cover: wooded savanna typically has 10%–30% tree canopy cover with a continuous grass layer, while mixed woodland with grass has 30%–60% tree canopy cover, with grasses forming the understory (Malan et al. 2007).

To assess the quality of the classifications for validation purposes, we calculated their overall accuracy and their Kappa coefficients with the following formula:

$$\text{Overall accuracy}=\frac{\;\text{Total number of correctly classified pixels}\;\left(\mathrm{TCP}\right)}{\text{Total number of reference pixels}\;\left(\mathrm{TRP}\right)}\;x\;100$$

This first operation gave us a map showing the different types of vegetation found in our study area. This map was then imported into R and, using the *terra* package (Hijmans et al. 2022), combined with the NDSM to create a new map with new classes. We aimed to create a more accurate map showing three vegetation types based on tree height (NDSM, Table 2).

**TABLE 2:** Vegetation type, based on tree height (according to NDSM).

Criterion: NDSM (m)	Vegetation type
NDSM < 1	Grassland
1 ≤ NDSM < 10	Shrubs and low trees
NDSM ≥ 10	Tall trees

#### 2.6.2 Assessment of Vulture Nest Presence With Respect to Vegetation Type and Tree Species

All quantitative data (height, circumference, canopy width, DTM, NDSM, slope, distance to river, distance to buildings and distance to roads) collected on nesting, control, and random trees were tested for normality using the Kolmogorov‐Smirnov test. All continuous variables failed the normality test (*n* = 2, *p* > 0.05) and so we applied nonparametric tests.

Since the expected numbers in some boxes are low (less than 5), we considered using a Fisher exact test rather than a chi‐square test (Nowacki 2017) to determine if there is an association between either of the categorical variables (vegetation type and tree species) and vulture nest presence.

#### 2.6.3 Comparison of Nesting Trees and Non‐Nesting Trees Characteristics

Since our continuous variables failed the normality test, we used the Kruskal–Wallis test to determine whether the variables differ between nesting and non‐nesting trees (Ostertagova et al. 2014). We then used the Dunn test for the pairwise multiple comparisons.

## 3 Results

### 3.1 Vegetation Types in Kempiana and Manyeleti Nature Reserves

**FIGURE 3:**
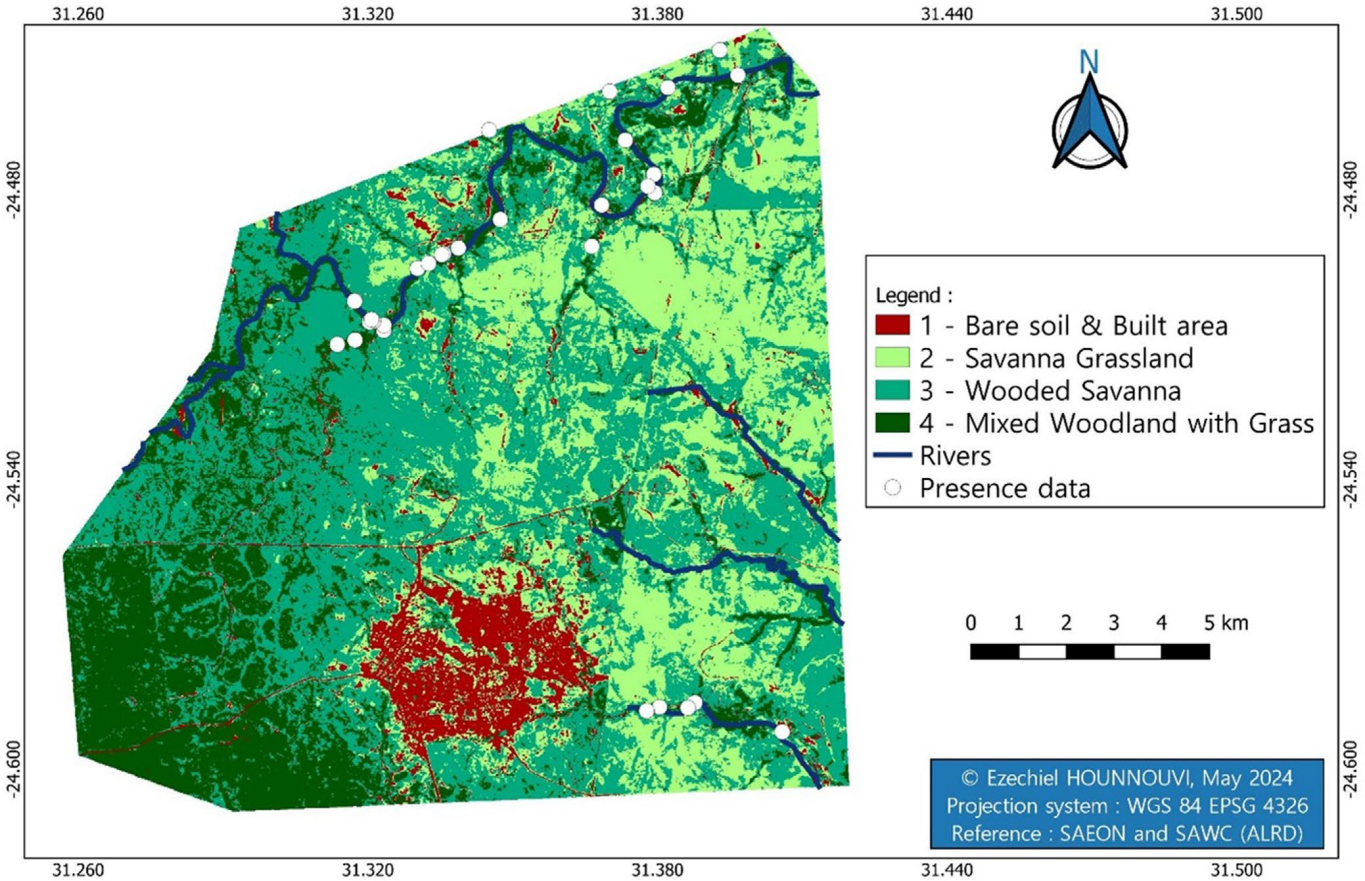
Vegetation types on Kempiana and Manyeleti Nature Reserves, north‐eastern South Africa.

The analysis of the aerial image shows the vegetation types in the study area. After processing, including vegetation type and tree height, four classes of vegetation were obtained (Figure 3).

The classification overall accuracy was 96.45%, and the Kappa coefficient (K) value obtained for image classification was 0.95.

The Kempiana and Manyeleti Reserves are made up of 59.1% wooded savanna, 19.9% savanna grassland, 15% Mixed woodland with grass, and 6% bare soil/built area.

The broad expanse of wooded savanna and savanna grassland had a variety of palatable grasses such as blue buffalo grass ( *Cenchrus ciliaris* ), finger grass ( *Digitaria eriantha* ) and stinking grass (*Bothriochloa radicans*). There were large tracts of grassland with stunted knob‐thorns (*Senegalia nigrescens*) punctuated by large trees, which were often restricted to the drainage lines. The most common trees in this vegetation habitat were the knob‐thorn, the umbrella thorn ( *Vachellia tortilis* ), marula ( *Sclerocarya birrea* ), jackalberry ( *Diospyros mespiliformis* ) and red bushwillow (*Combretum apiculatum*).

In the case of mixed woodland with grass, these are in fact the trees of riparian forests, including the sycamore fig ( *Ficus sycomorus* ), leadwood ( *Combretum imberbe* ), jackalberry, Natal mahogany ( *Trichilia emetica* ), tamboti (*Spirostachys africana*), weeping boer‐bean (*Schotia brachypetala*), apple‐leaf (*Philenoptera violacea*) and nyala berry (*Xanthocercis zambesiaca*). Mixed deciduous forests are the vegetation types found along riverbanks. The main trees are bushwillow or *Combretum* species—red bushwillow (*Combretum apiculatum*), russet bushwillow ( *Combretum hereroense* ), as well as large marulas ( *Sclerocarya birrea* ), magic guarri (*Euclea divinorum*), pockets of knob thorn (*Senegalia nigrescens*) and round‐leaved bloodwood (*Pterocarpus rotundifolius*), also known as teak.

### 3.2 Nest Placement With Respect to Vegetation Habitat

**FIGURE 4:**
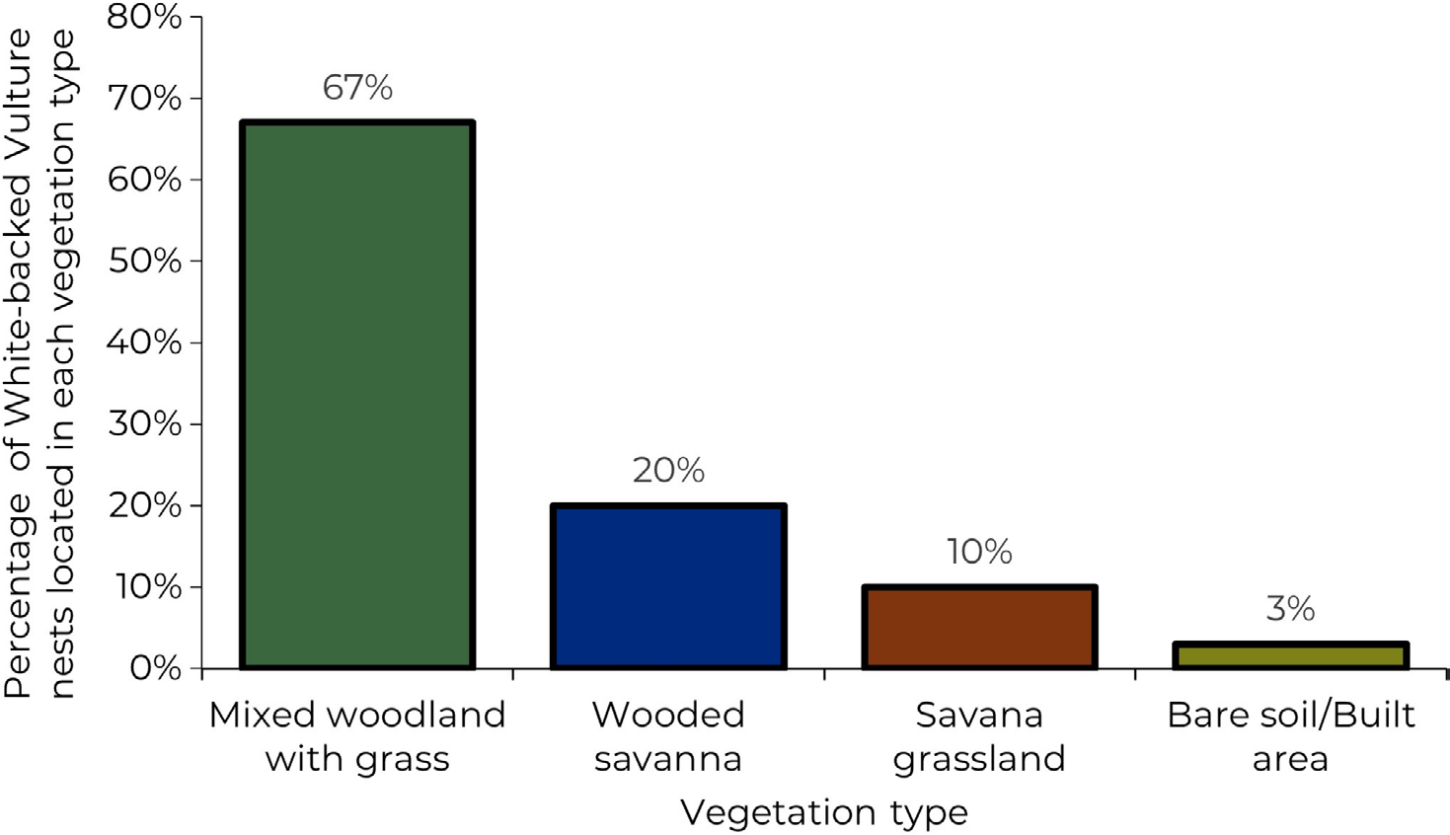
Vulture nest placement with respect to vegetation type in Kempiana and Manyeleti Nature Reserves.

All White‐backed Vulture nests that we found in this study were located near water bodies, either along rivers or near pans. Three vegetation communities were determined in addition to the built area class, and of 106 trees surveyed, 31 were vulture nesting trees. Of the four classes determined, we found nesting trees in all these classes (Figure 4).

As we can observe, 67% of nests were found in mixed woodland with grass vegetation, and Fischer's exact test shows us that there was a significant association between nest presence and vegetation types (*F* = 92.3, *p* < 0.05).

### 3.3 Selection of Tree Species for Nest Placement

Of the 19 tree species surveyed, all 31 nests were found in just eight tree species: 21 nests were in *Diospyros mespiliformis* , 03 in *Phylenoptera violacea*, 02 in *Schotia brachypetala*, and 01 each in *Combretum imberbe* , *Ficus sycomorus, Senegalia negrescens*, and *Spyrostachys africana*.

**FIGURE 5:**
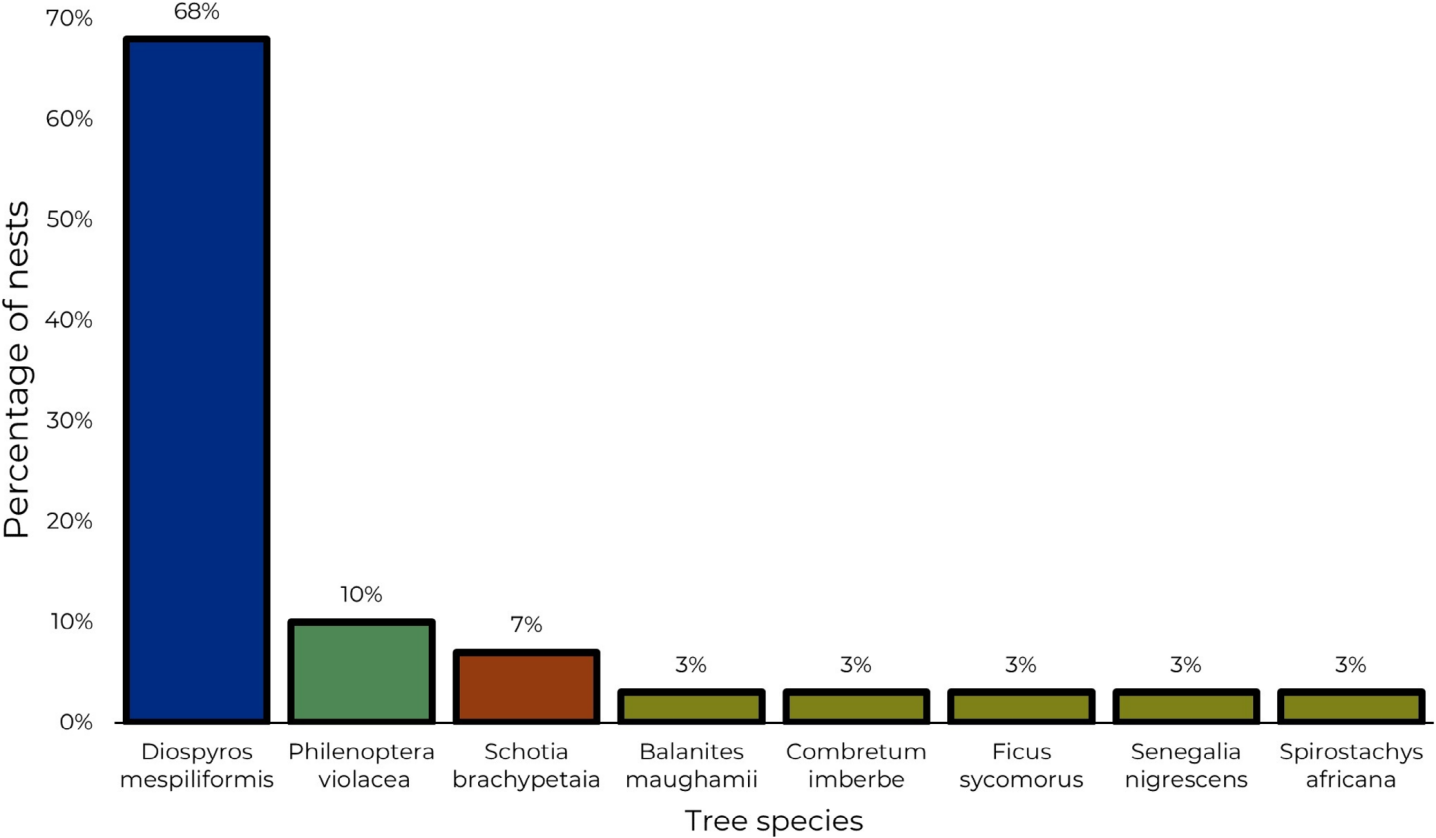
White‐backed Vulture nest presence with respect to nesting tree species in Kempiana and Manyeleti Nature Reserves.

*Diospyros mespiliformis* was the tree species that was most frequently used by the White‐backed Vulture for their nest placement (Figure 5). We found more nests in *Diospyros mespiliformis* (68%) than in the other seven tree species combined (32%). There was a significant association between nest presence and tree species (F = 78.5, *p* < 0.05).

### 3.4 Comparison Between Nest Sites and Random Site Characteristics

We have compared two groups of trees (nesting trees and non‐nesting trees) trees, (Table 3).

**TABLE 3:** Comparison of characteristics of nesting and non‐nesting trees. The values are presented as median ± SD.

Tree characteristics	Nesting trees (*n* = 31)	Non‐nesting trees (*n* = 75)
Height (m)	15 ± 3.6	13 ± 3.7
CBH 1 (cm)	267 ± 84	230 ± 116
CBH 2 (cm)	304 ± 103	242 ± 100
Canopy width (m)	16.2 ± 4.0	13.0 ± 4.3
DTM (m)	440.8 ± 9.7	433.9 ± 12.4
Distance_to_Rivers (km)	1.4 ± 4.9	2.0 ± 10.1
Distance_to_Roads (km)	5.6 ± 10.9	3.8 ± 14.1
Slope (°)	2.8 ± 1.9	1.6 ± 1.7
Distance_to_building (km)	13.5 ± 8.9	7.3 ± 5.6

For a deeper understanding of differences between characteristics of the three groups of trees, a Kruskal‐Wallis test showed there are significant differences between the two groups with regard to height, CBH 1, CBH 2, Distance_to_Rivers, and Distance_to_Roads (*p* < 0.05) (Table 4). Canopy width, DTM, slope, and Distance_to_buildings did not appear to be important for nest selection for the White‐backed Vulture (*p* ≥ 0.05).

**TABLE 4:** Kruskal‐Wallis test results when comparing variables for the three groups: Vulture nest trees and random trees in Kempiana and Manyeleti Nature Reserves.

Variables	Test Statistic	*p*	Significance
Height	9.43	0.00 < 0.01	**
CBH1	7.03	0.00 < 0.01	**
CBH2	8.27	0.00 < 0.01	**
Canopy width	3.06	0.07 > 0.05	ns
DTM	0.92	0.33 > 0.05	ns
Distance_to_Rivers	4.19	0.03 < 0.05	*
Distance_to_Roads	5.29	0.02 < 0.05	*
Slope	0.18	0.66 > 0.05	ns
Distance_to_building	0.62	0.42 > 0.05	ns

* *p* < 0.05 significant.

** *p* < 0.01 very significant.

*** *p* < 0.001 extremely significant.

**FIGURE 6:**
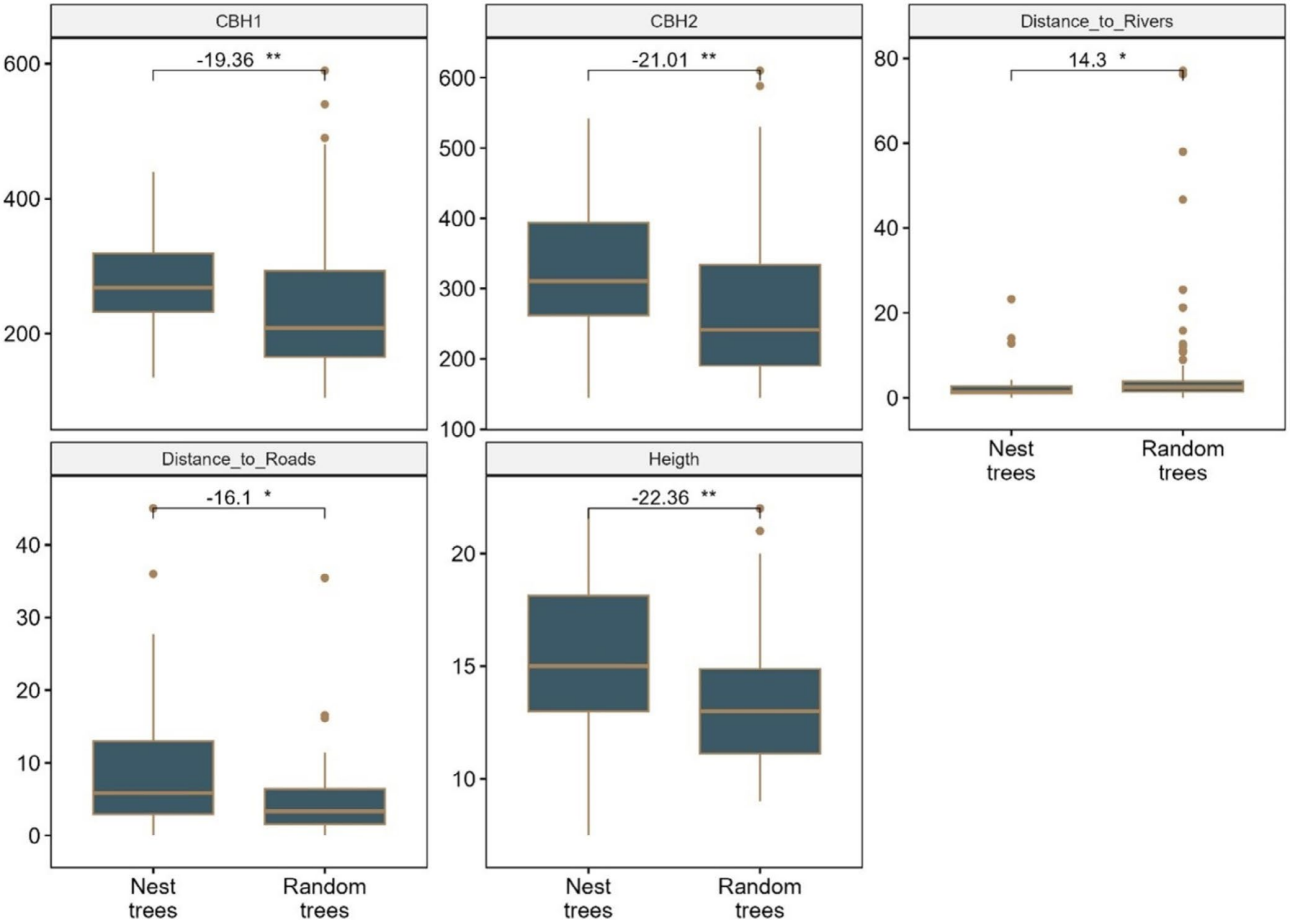
A comparison of various parameters for White‐backed Vulture nest trees and random trees.

As the results of the Kruskal–Wallis test were statistically significant for some variables, we performed the Dunn test to determine where the differences lay at the 5% threshold between the nest trees group and non‐nesting trees (random trees). The graphs below show the differences after applying the Dunn test (Figure 6).

### 3.5 Nesting Tree vs. Random Trees

The Dunn test showed that there were significant differences between nesting and non‐nesting trees (random trees) for five variables (Height, CBH 1, CBH 2, Distance to_rivers and Distance_to_Roads). In this case, when considering median values, nesting trees are taller than non‐nesting trees (15 m vs. 13 m), have a significantly larger CBH than random trees (304 cm vs. 242 cm), and are also significantly farther to roads (5.6 km vs. 3.8 km) and significantly closer to rivers than random trees (1.4 km vs. 2 km;).

## 4 Discussion

In Kempiana and Manyeleti Nature Reserves in north‐eastern South Africa, White‐backed Vulture nest trees were significantly taller, with significantly larger CBH, and were significantly closer to rivers and to roads. Most nests (68%) were located on *Diospyros mespiliformis* , and there was a significant association between vulture nest presence and tree species.

### 4.1 Vegetation Types in Kempiana and Manyeleti Nature Reserves

The study area was dominated by savanna, followed by patches of grassland and woodland. Our results are similar to those of Du Toit et al. (2003) who described the vegetation of the region as covered by lowveld savanna and woodland with grass areas underlain by granite. Mucina and Rutherford (2006) found similar results in their study on the vegetation of South Africa, Lesotho, and Eswatini. The authors showed that two types of biomes (grassland and savanna) covered the province of Mpumalanga.

### 4.2 Vulture Nest Presence With Respect to Vegetation Type

The vegetation analysis in Kempiana and Manyeleti Nature Reserves shows that White‐backed Vulture nests were recorded in several vegetation habitat types (savanna grassland, wooded savanna, woodland and even in an anthropized environment). This supports Mundy (1982) who reported that White‐backed Vulture was recorded in a wide variety of vegetation types.

We found that this species' preference in terms of nesting sites was woodland or riverine woodland. This is because the mixed woodland with soft grass and wooded savanna are the vegetation habitats in Kempiana and Manyeleti where tall trees occur, which can accommodate raptor nests. Chomba and M'Simuko (2013) found the same result in their study on the nesting patterns of White‐backed Vultures and African Fish Eagles ( *Haliaeetus vocifer* ) in Lochinvar National Park, Zambia. They remarked that knob‐thorn and Mopane woodland contained tall trees and that is why they recorded > 80% of nesting trees in these vegetation types during their surveys. The higher number of nesting trees found in wooded savanna and mixed woodland with grass can be attributed to the presence of large, mature trees that thrive in these areas near rivers, where the environment provides optimal conditions for their growth and suitability for vulture nests.

The White‐backed Vulture's absence from built areas and savanna grassland is probably due to the lack of tall trees in those vegetation habitats. A study by Monadjem and Garcelon (2005) on the nesting distribution of vultures in relation to land use in Eswatini showed that White‐backed Vultures nested almost exclusively in riparian vegetation at all sites except at Hlane National Park. Results from the survey of Mundava et al. (2018) in Hwange National Park, Zimbabwe, also confirmed that White‐backed Vultures nest in riverine habitats with nests mostly placed at the tops of tall trees. Thus, venturing farther from riverine vegetation, which contains the tallest trees, decreases the likelihood of finding suitable nesting trees, resulting in a lower density of nests (Virani et al. 2011).

### 4.3 Selection of Tree Species for Nest Placement

The tree species in which White‐backed Vulture nests were most frequently located in our study was *D. mespiliformis* . This species was also reported as the most used tree for nesting White‐backed Vultures in South Africa by Mundy (1982). If this tree species is the most used by White‐backed Vultures for nesting, it is likely because of its structure (Monadjem et al. 2016). *Diospyros mespiliformis* has a dense, leafy canopy that effectively conceals White‐backed Vulture nests from predators on the ground. The median height of nest trees in this study (16 m) was slightly lower than that reported for nests in Zimbabwe (18.1 m) by Mundy (1982). However, preference for, or avoidance of, a particular tree cannot be determined merely by recording the proportion of nests placed in different species of trees. The availability of the various tree species must also be considered. *Diospyros mespiliformis* is generally abundant in different habitats in our study area even if the nests on the river towards the west are mainly knob‐thorns. In many areas, it might be the only suitable tree (in terms of height and especially in terms of canopy density) available to vultures. Monadjem et al. (2016) reported the same observation at Olifants River Private Nature Reserve, Limpopo Province in South Africa, where they found that *Diospyros mespiliformis* was the tree species most used by vultures for nesting because these trees were abundant. The vultures might have used this species due to its ability to support their massive nests and their combined body mass. *Diospyros mespiliformis* has high and large spreading branches, which could help maximize nest height and may, in turn, provide a better vantage point and reduce accessibility for mammalian predators (Monadjem et al. 2016).

Most texts usually include *Senegalia nigrescens* as preferred nesting sites of White‐backed Vultures (Mundava et al. 2018). In contrast, we found that only 3% of White‐backed Vulture nests were located in trees of this Genus in our study area, and most of these trees seemed unhealthy (Gandaho, unpublished data, 2024), having been debarked and destroyed by elephants. Most of these trees also had insect damage due to the debarking caused by elephants. This could explain the fact that vultures nest in *D. mespiliformis* to avoid nesting in damaged knob‐thorns that would potentially not support their nests for a long time. These conclusions correspond with the findings of Monadjem and Garcelon (2005) who found that White‐backed Vultures do not nest in areas where there is severe elephant impact on trees. One of the limitations of our study is that it was carried out only along rivers, and *D. mespiliformis* is closely associated with rivers.

### 4.4 Comparison of Nesting Trees With Random Trees

The African vulture does not choose trees at random to build its nest. It selects trees that have certain precise characteristics, such as being tall, having a large trunk circumference, and being located in dense vegetation near rivers (Anoop et al. 2020; Virani et al. 2011). These are the points of difference between nesting trees and non‐nesting trees. Our study, therefore, makes a significant contribution to the understanding of White‐backed Vulture nest tree selection, providing insights into the characteristics of preferred nesting sites. The characteristics of random trees are a little similar to those of nesting trees based on the minimum criteria of nesting trees (height and trunk circumference) and so random trees are more likely to be suitable for White‐backed Vulture nesting; however, they were not used for nesting. A possible explanation is that apart from simply selecting a taller tree, perhaps the most important nest‐site characteristic of all for a White‐backed Vulture is the presence of other breeding vultures nearby or the connection between these trees and rivers. Many other authors, like Cortes‐Avizanda et al. (2014), Harel et al. (2017), Mateo‐Tomás and Olea (2011) and Kendall et al. (2012), confirmed that the social imperatives of group living may have a greater impact on vulture nest‐site selection than the actual nest trees due to the benefits of social foraging and using social cues for breeding site selection.

The fact that the nesting trees are farther from the roads can be explained by the fact that the two reserves in our study area are considered to be tourist areas where sightseeing is of paramount importance. As a result, many small roads have been created, especially around river lines, since there are more animals to see along the rivers. Large groups of people, along with noise from vehicle exhausts and honking, would disrupt the birds (Chomba and M'Simuko 2013).

In conclusion, our findings are consistent with the studies of Kemp and Kemp (1975) and Monadjem (2003), who proposed that African White‐backed Vultures exhibit selective preferences for specific tree species when choosing nesting sites. We advocate for the implementation of localized land management strategies focused on preserving tall trees in proximity to riverine areas, both within and surrounding extant African White‐backed Vulture colonies, to ensure the conservation of critical nesting habitats.

Additionally, future studies could further explore these aspects by investigating nest clustering patterns, conducting behavioral observations, or utilizing tracking data to evaluate social interactions among breeding vultures. Assessing nest success rates could help identify which nest sites contribute most significantly to vulture reproduction. Long‐term monitoring of vulture nests in the Kempiana and Mayeletti reserves is essential to assess the impacts of elephants and fire damage on trees and their consequent effects on nesting success. Continuous surveillance of nests and fledglings would yield more accurate estimates of breeding success, a critical demographic parameter for effectively assessing the conservation status and population dynamics of the African White‐backed Vulture.

## Author Contributions

**Fidèle Ezéchiel K. Hounnouvi:** conceptualization (equal), formal analysis (equal), investigation (equal), methodology (equal), visualization (equal), writing – original draft (equal), writing – review and editing (equal). **Stanislas Mahussi Gandaho:** data curation (equal). **Fern Bain:** data curation (equal). **Peter Hamming:** conceptualization (equal), investigation (equal), methodology (equal), supervision (equal), writing – review and editing (equal). **Lindy J. Thompson:** conceptualization (equal), methodology (equal), supervision (equal), writing – review and editing (equal).

## Ethics Statement

The authors have nothing to report. Our fieldwork was purely observational. We conducted our fieldwork outside of the vulture breeding season, and so the nests that we encountered were not yet in use. No vultures were disturbed during our fieldwork.

## Conflicts of Interest

The authors declare no conflicts of interest.

## Data Availability

Aerial image: https://doi.org/10.15493/SAEON.NDLOVU.220424. Digital Terrain Model: https://doi.org/10.15493/SAEON.NDLOVU.220424. Normalized Digital Surface Model: https://doi.org/10.15493/SAEON.NDLOVU.220424. Slope: https://doi.org/10.5069/G9028PQ. Distance to River: https://download.bbbike.org/osm/extract/planet_30.89,‐24.886_31.746,‐24.287.osm.shp.fr.zip. Distance to Road: https://download.bbbike.org/osm/extract/planet_30.89,‐24.886_31.746,‐24.287.osm.shp.fr.zip. Distance to Building: https://download.bbbike.org/osm/extract/planet_30.89,‐24.886_31.746,‐24.287.osm.shp.fr.zip.
